# PEDV Nsp14 induces mitophagy-mediated degradation of MAVS to antagonize host innate immunity and facilitate viral proliferation

**DOI:** 10.1128/jvi.00498-25

**Published:** 2025-07-09

**Authors:** Lei Yang, Qisheng Qian, Yan-gang Sun, Xin-xin Chen, Guangxu Xing, Jia-qing Zhang, Bao-song Xing, Songlin Qiao, Rui Li, Gaiping Zhang

**Affiliations:** 1 College of Veterinary Medicine, Henan Agricultural University 731518, Zhengzhou, Henan, China; 2 Institute for Animal Health, Henan Academy of Agricultural Sciences 74728, Zhengzhou, Henan, China; 3 Longhu Laboratory 693032 , Zhengzhou, Henan, China; 4 Henan University of Chinese Medicine 232830, Zhengzhou, Henan, China; 5 Institute of Animal Husbandry, Henan Academy of Agricultural Sciences 74728, Zhengzhou, Henan, China; University of North Carolina at Chapel Hill, Chapel Hill, North Carolina, USA

**Keywords:** PEDV, Nsp14, mitophagy, IFN-β, NDP52

## Abstract

Porcine epidemic diarrhea virus (PEDV) leads to a high mortality in neonatal piglets and causes serious harm to the global swine industry. PEDV has been shown to exploit diverse strategies for antagonism of host innate immunity and promotion of self-replication. However, the underlying mechanisms involved in PEDV immunosuppression remain to be fully elucidated. The current study reveals that PEDV triggers mitophagy to suppress host innate immune responses and facilitate viral proliferation. Mechanistically, PEDV non-structural protein (Nsp) 14 was identified to mediate the interaction between the mitophagy receptor NDP52 and mitochondrial outer membrane protein TOM20 to induce mitophagy. Subsequently, Nsp14-induced mitophagy resulted in the degradation of mitochondrial antiviral signaling protein (MAVS) to suppress interferon-β (IFN-β) production and promote viral propagation. These findings deepen the understanding of PEDV pathogenesis and provide novel targets for the development of antiviral avenues.

**IMPORTANCE:**

The global pig farming industry has suffered huge economic losses from PEDV, underscoring an urgent need for in-depth research on its pathogenesis. Host innate immunity functions as the first line of defense against PEDV propagation, and PEDV has developed multiple countermeasures to dampen host antiviral responses. Here, we found that PEDV Nsp14 induced mitophagy via mediating the interaction between NDP52 and TOM20, which led to MAVS degradation and hampered IFN-β production. Therefore, our work unveils a novel mechanism by which PEDV antagonizes host innate immunity to facilitate its proliferation and is beneficial for the prevention and control of the virus.

## INTRODUCTION

Porcine epidemic diarrhea (PED), caused by PED virus (PEDV), is a highly contagious disease with characteristic symptoms including vomiting, dehydration, and watery diarrhea (1, 2). PED affects pigs of all ages, and especially its mortality in suckling piglets reaches 100% (3). PED re-emerged in China in 2010 (4) and spread widely in the United States and other pig-raising countries, causing serious economic losses to the global pig industry (5, 6).

PEDV is a single-stranded positive-sense RNA virus belonging to the *Alphacoronavirus* genus of the *Coronaviridae* family (7). Its genome is approximately 28 kb in length (8) and encodes 16 non-structural proteins (Nsps, Nsp1-16), spike (S) glycoprotein, membrane (M) protein, envelope (E) protein, nucleocapsid (N) protein, and an accessory protein known as open reading frame 3 (ORF3) (9, 10). PEDV infects the intestinal epithelial cells of pigs (11, 12) and their derived cell lines, for example, IPEC-J2 (13). In addition, it is capable of infecting African green monkey kidney cells Vero (14).

Innate immunity is the first line of defense against RNA virus infections in hosts (15). During PEDV infection, retinoic acid-inducible gene I (RIG-I)-like receptors (RLRs), such as melanoma differentiation-associated gene 5 (MDA5) and RIG-I, detect viral RNA and activate the mitochondrial antiviral signaling protein (MAVS)-TANK-binding kinase 1 (TBK1)-interferon regulatory factor 3 (IRF3) signaling pathway. This activation leads to the production of type I interferons (IFN-I), such as IFN-α and IFN-β, for the establishment of a cellular antiviral state (16). However, PEDV has been shown to utilize various strategies for suppressing host innate immune responses, which poses challenges in controlling and preventing this virus (17). Despite much research, the underlying mechanisms involved in PEDV immunosuppression remain incompletely understood.

Mitochondria are the powerhouses of energy production in eukaryotic cells (18). In addition, they have gained increasing attention as the platforms for innate immune signaling because MAVS is anchored on their outer membranes and participates in antiviral signal transduction (19, 20). Excessive or damaged mitochondria undergo selective autophagy, namely mitophagy, to ensure their homeostasis within the cells (21, 22). There are several studies indicating that viruses induce mitophagy to degrade MAVS, which impairs host innate immunity and promotes viral replication (23–26).

In this study, we initially validated the inhibition of IFN-β production by PEDV Nsp14. We subsequently found that PEDV Nsp14 triggered mitophagy to degrade MAVS and dissected the detailed mechanisms. We next determined that Nsp14-induced MAVS degradation via mitophagy dampened innate immunity responses and facilitated PEDV propagation.

## RESULTS

### PEDV Nsp14 inhibits IFN-β production

**Fig 1:**
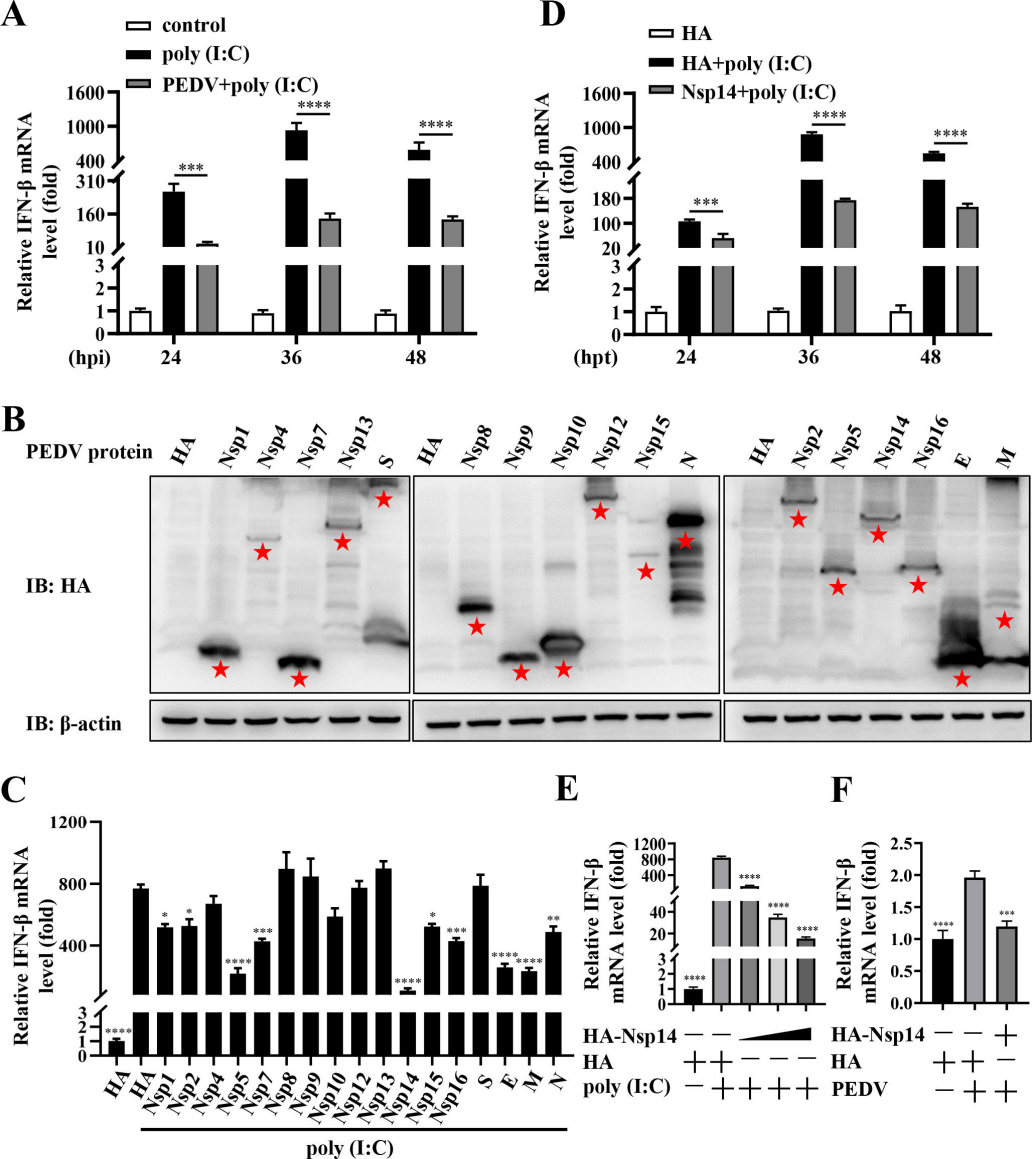
PEDV Nsp14 inhibits IFN-β production. (**A**) IPEC-J2 cells were infected with or without 0.2 multiplicity of infection (MOI) PEDV, and then transfected with 5 µg/mL poly (I:C) at 18 hpi. The cells were collected at 24, 36, and 48 hpi, and the IFN-β mRNA levels were evaluated by RT-qPCR. The cells without PEDV infection and poly (I:C) transfection served as a negative control. (**B**) Each HA-tagged PEDV protein expression vector and HA-tagged empty vector (the pCAGGS-HA plasmid) were transfected separately into IPEC-J2 cells. After transfection for 36 h, protein expression was analyzed using WB. Asterisks marked the target proteins. (**C**) Each HA-tagged PEDV protein expression vector and HA-tagged empty vector were transfected separately into IPEC-J2 cells for 36 h. Following poly (I:C) transfection, RT-qPCR was used to assess IFN-β mRNA levels. The cells transfected with the HA-tagged empty vector alone served as a negative control. (**D**) IPEC-J2 cells were transfected with either the pCAGGS-HA-Nsp14 plasmid or HA-tagged empty vector. After transfection for 18 h, the cells were transfected with or without 5 µg/mL poly (I:C). The cells were collected at the indicated time points (24, 36, and 48 hpt). The IFN-β mRNA levels were assessed using RT-qPCR. (**E**) IPEC-J2 cells were transfected with gradient concentrations of the pCAGGS-HA-Nsp14 plasmid (0.5, 1, and 2 µg) or HA-tagged empty vector, followed by poly (I:C) transfection, and RT-qPCR was used to assess IFN-β mRNA levels. (**F**) IPEC-J2 cells were transfected with either the pCAGGS-HA-Nsp14 plasmid or HA-tagged empty vector. At 24 hpt, the cells were infected with or without 0.4 MOI PEDV for an additional 12 h. The IFN-β mRNA levels were assessed using RT-qPCR. The data from three independent experiments were presented as means ± SEM. Statistical analysis was conducted with one-way ANOVA or Student’s *t* test. *P < 0.05, **P < 0.01, ***P < 0.001, and ****P < 0.0001.

As IFN-I triggers potent defense against viruses (27), we initially utilized quantitative real-time polymerase chain reaction (RT-qPCR) to assess PEDV inhibition of IFN-β production at 24, 36, and 48 h post-infection (hpi). As shown in Fig. 1A, the mRNA levels of IFN-β induced by RNA analogue poly (I:C) were significantly decreased in the PEDV-infected IPEC-J2 cells, and the reduction was most pronounced at 36 hpi, indicating that PEDV infection hampered IFN-β production. To identify which PEDV proteins were responsible for this suppression, we successfully expressed its Nsp1, 2, 4, 5, 7-10, 12-16, S, M, E, and N proteins with hemagglutinin (HA) tag (Fig. 1B) and found that Nsp1, 2, 5, 7, 14-16, E, M, and N suppressed IFN-β production (Fig. 1C). Among them, Nsp14 showed the most remarkable suppressive effect (Fig. 1C). As the mechanisms involved in its suppressive function have not been clearly clarified (13, 28, 29), we chose PEDV Nsp14 for further exploration. HA-Nsp14 was shown to exhibit the strongest inhibitory effect on IFN-β production at 36 h post-transfection (hpt; Fig. 1D) and inhibit its production in a dose-dependent manner (Fig. 1E). In addition, HA-Nsp14 inhibited PEDV-induced IFN-β production (Fig. 1F). These results confirm that PEDV Nsp14 acts as a suppressor of IFN-β production.

### PEDV Nsp14 inhibits IFN-β production by degrading MAVS via the autolysosomal pathway

**Fig 2:**
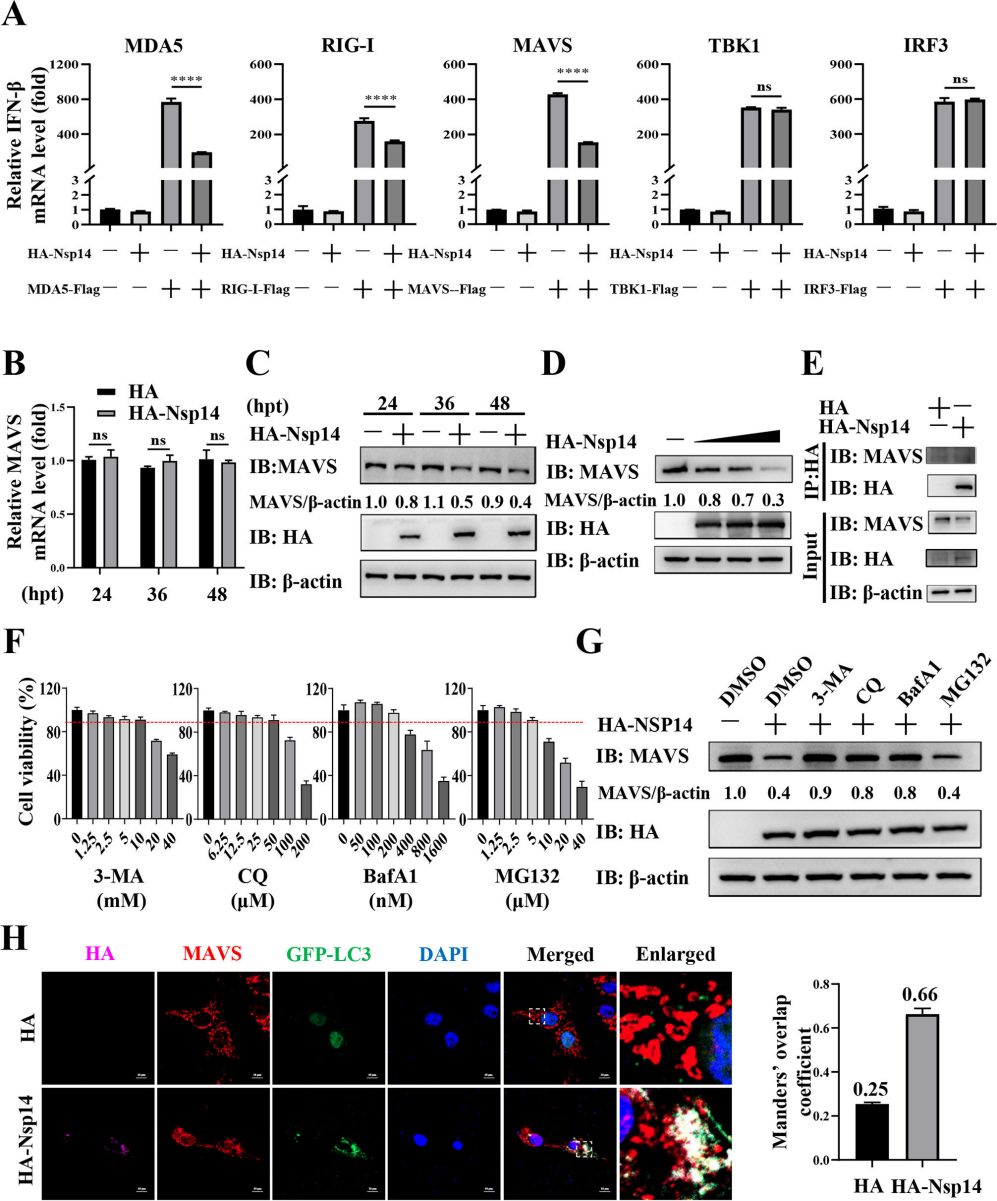
PEDV Nsp14 inhibits IFN-β production by degrading MAVS via the autolysosomal pathway. (**A**) IPEC-J2 cells were co-transfected with either the pCAGGS-HA-Nsp14 plasmid or HA-tagged empty vector, and the Flag-tagged empty vector (the pCMV-3 × Flag-Neo plasmid), MDA5-Flag, RIG-I-Flag, MAVS-Flag, TBK1-Flag, or IRF3-Flag plasmid for 36 h. Subsequently, the levels of IFN-β mRNA were assessed using RT-qPCR. The cells co-transfected with HA-tagged empty vector and the Flag-tagged empty vector served as controls. (**B**) and (**C**) IPEC-J2 cells were transfected with either the pCAGGS-HA-Nsp14 plasmid or HA-tagged empty vector. The cells were collected at the indicated time points (24, 36, and 48 hpt). The MAVS mRNA and protein levels were detected by RT-qPCR and WB, respectively. (**D**) IPEC-J2 cells were transfected with the HA-tagged empty vector and gradient concentrations of pCAGGS-HA-Nsp14 plasmid (0.5, 1, and 2 µg) for 36 h and analyzed using WB. (**E**) IPEC-J2 cells were transfected with the pCAGGS-HA-Nsp14 plasmid or HA-tagged empty vector. After 36 h of transfection, the IP assay was performed with anti-HA magnetic beads, and WB was conducted with the specific antibodies. (**F**) The cytotoxicity of 3-MA, CQ, BafA1, or MG132 on IPEC-J2 cells was detected using the cell viability assay. (**G**) The pCAGGS-HA-Nsp14 plasmid or HA-tagged empty vector was transfected into IPEC-J2 cells. At 6 hpt, the cells were treated with 3-MA (10 mM), CQ (50 µM), BafA1 (200 nM), or MG132 (5 µM) for an additional 30 h. WCLs were analyzed by WB. (**H**) IPEC-J2 cells were co-transfected with the GFP-LC3 plasmid and the pCAGGS-HA-Nsp14 plasmid or HA-tagged empty vector. At 6 hpt, the cells were treated with CQ for an additional 30 h. The cells were subjected to confocal microscopy using the specific antibodies and fluorescent reagents. The assessment of co-localization was conducted by calculating Manders’ overlap coefficient using the JaCoP plugin in the ImageJ software. Scale bars = 10 µm. Data represent means ± SEM from three independent experiments. Statistical analysis was carried out using Student’s *t* test. ns *P >* 0.05 and ****P < 0.0001.

To elucidate the mechanism by which PEDV Nsp14 inhibited IFN-β production, we examined its targets involved in the RLR signaling pathway by assessment of IFN-β mRNA levels after HA-Nsp14 was co-transfected with MDA5-Flag, RIG-I-Flag, MAVS-Flag, TBK1-Flag, or IRF3-Flag in IPEC-J2 cells. As shown in Fig. 2A, the IFN-β production was elevated by expression of each RLR signaling pathway protein. The elevated IFN-β mRNA level was unaffected by HA-Nsp14 in the TBK1-Flag or IRF3-Flag-overexpressed cells, while it was lowered by HA-Nsp14 in the MDA5-Flag, RIG-I-Flag, or MAVS-Flag-overexpressed ones, which suggested that PEDV Nsp14 targeted MDA5, RIG-I, and MAVS. There have been reports showing that PEDV modulates MDA5 dephosphorylation (30) and RIG-I ubiquitination (31), and, therefore, we focused on MAVS in the current study. Subsequently, we detected the effect of HA-Nsp14 on MAVS mRNA and protein levels, respectively. RT-qPCR analyses showed that Nsp14 overexpression took no effect on MAVS mRNA abundance at different time points (Fig. 2B). By contrast, Western blot (WB) analysis disclosed that its overexpression decreased the MAVS protein levels in a time- and dose-dependent manner (Fig. 2C and D). These findings indicate that PEDV Nsp14 suppresses IFN-β production by degrading MAVS.

We subsequently explored how PEDV Nsp14 degraded MAVS. As shown in Fig. 2E, Nsp14 did not interact with MAVS, excluding that Nsp14 itself degraded MAVS. As the autolysosomal pathway and ubiquitin-proteasome system are two primary manners for protein degradation (32), we applied the autophagy inhibitor 3-methyladenine (3-MA), the lysosomal inhibitor chloroquine (CQ) and bafilomycin A1 (BafA1), and the proteasome inhibitor MG132 to identify the specific one involved in MAVS degradation by Nsp14. To determine the non-cytotoxic concentration of each inhibitor, we performed cell viability assays and selected 10 mM for 3-MA, 50 µM for CQ, 200 nM for BafA1, and 5 µM for MG132 for subsequent experiments (Fig. 2F). We observed that the Nsp14-mediated degradation of MAVS was impeded by 3-MA, CQ, and BafA1, which suggested that Nsp14 degraded MAVS dependent on the autolysosomal pathway (Fig. 2G). In addition, we monitored the co-localization of MAVS with the autophagy marker GFP (green fluorescent protein)-LC3 (microtubule-associated protein light chain 3) in the Nsp14-overexpressed cells (Fig. 2H). Manders’ overlap coefficient analysis also showed the increased co-localization between MAVS and GFP-LC3 (Manders’ overlap coefficient increased from 0.25 to 0.66, Fig. 2H). These data show that PEDV Nsp14 degrades MAVS via the autolysosomal pathway.

### PEDV Nsp14 induces mitophagy

**Fig 3:**
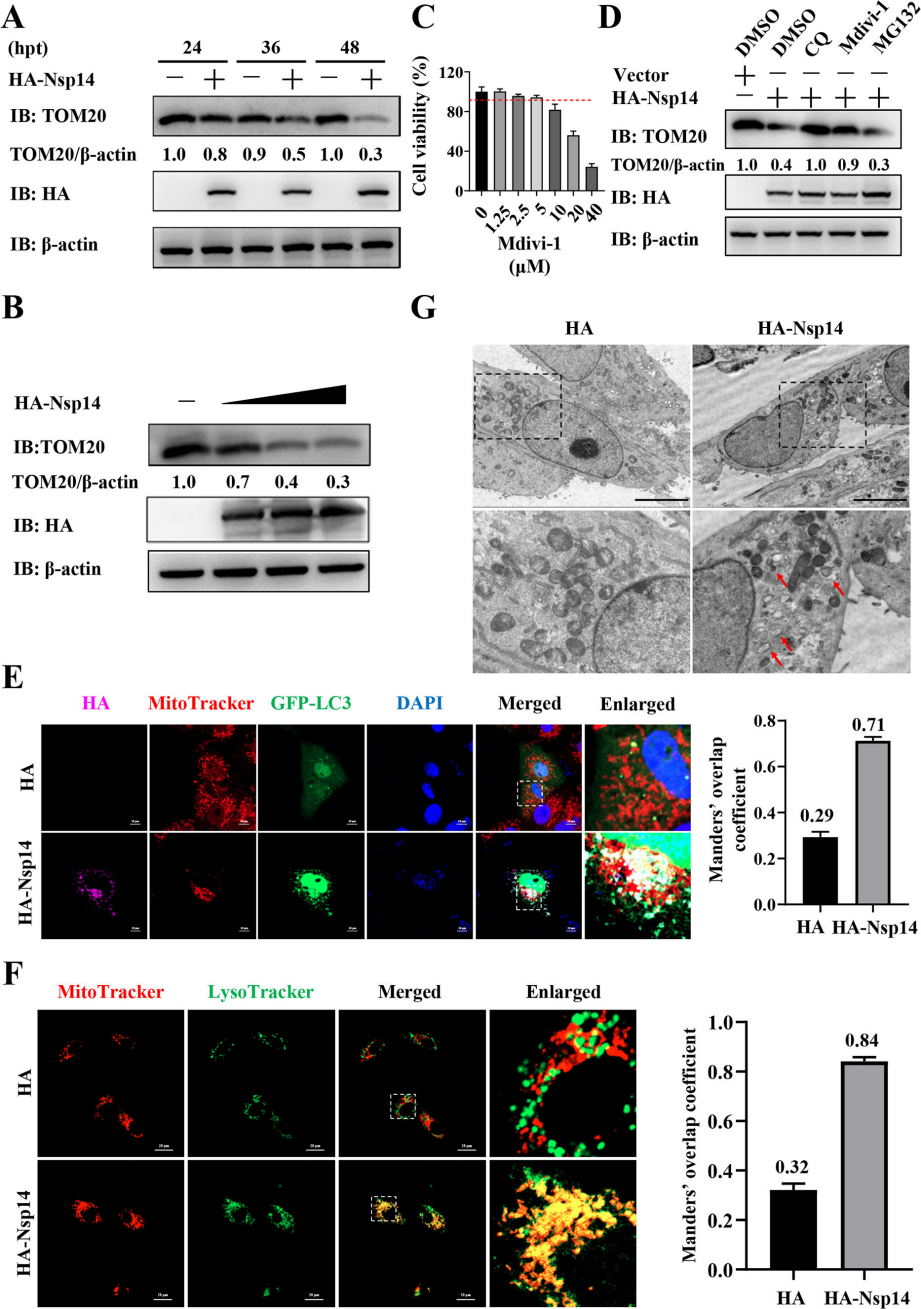
PEDV Nsp14 induces mitophagy. (**A**) The pCAGGS-HA-Nsp14 plasmid or HA-tagged empty vector was transfected into IPEC-J2 cells. WCLs were prepared at 24, 36, and 48 hpt for WB analysis. (**B**) IPEC-J2 cells were transfected with the pCAGGS-HA-Nsp14 plasmid (0.5, 1, and 2 µg) or HA-tagged empty vector. WCLs were prepared at 36 hpt for WB analysis. (**C**) The cytotoxicity of Mdivi-1 on IPEC-J2 cells was detected using the cell viability assay. (**D**) IPEC-J2 cells were transfected with the pCAGGS-HA-Nsp14 plasmid or HA-tagged empty vector for 6 h, followed by treatment with CQ (50 µM), Mdivi-1 (5 µM), or MG132 (5 µM) for 30 h. The WCLs were subjected to WB analysis. (**E**) IPEC-J2 cells were transfected with the GFP-LC3 plasmid along with HA-tagged empty vector or pCAGGS-HA-Nsp14 plasmid for 36 h. The cells were subjected to confocal microscopy using the specific antibodies and fluorescent reagents. The assessment of co-localization was conducted by calculating Manders’ overlap coefficient using the JaCoP plugin in the ImageJ software. Scale bars = 10 µm. (**F**) The pCAGGS-HA-Nsp14 plasmid or HA-tagged empty vector was transfected into IPEC-J2 cells for 36 h. The cells were stained using MitoTracker (red) and LysoTracker (green). The fluorescence signals were visualized in live cells using confocal microscopy. The assessment of co-localization was conducted by calculating Manders’ overlap coefficient using the JaCoP plugin in the ImageJ software. Scale bars = 20 µm. (**G**) IPEC-J2 cells were transfected with the pCAGGS-HA-Nsp14 plasmid or HA-tagged empty vector. Mitochondrial morphology was detected by TEM (scale bars = 5 µm). Red arrows indicate the mitochondria engulfed by autophagosomes.

Previous studies have reported viral induction of mitophagy, a selective autophagy, for degrading mitochondria-localized MAVS and suppressing IFN-β synthesis (33). Consequently, we hypothesized that PEDV Nsp14 degraded MAVS via mitophagy and investigated whether it triggered mitophagy in IPEC-J2 cells. We initially assessed the impact of Nsp14 on TOM20 abundance, a mitochondrial outer membrane protein as a marker for mitochondria (34). WB analyses revealed that Nsp14 decreased TOM20 abundance in a time- and dose-dependent manner (Fig. 3A and B). However, Mdivi-1, a mitophagy inhibitor (35), prevented Nsp14-induced decrease in TOM20 abundance at a non-toxic concentration of 5 µM, suggesting that Nsp14 induced mitophagy (Fig. 3C and D). To further validate Nsp14-induced mitophagy, we overexpressed GFP-LC3 and HA-Nsp14 and labeled mitochondria with the specific mitochondrial fluorescent staining dye MitoTracker Red CMXRos (referred to as MitoTracker). Confocal microscopy showed the co-localization between GFP-LC3 and mitochondria in the Nsp14-overexpressed cells (Manders’ overlap coefficient increased from 0.29 to 0.71, Fig. 3E), indicating that Nsp14 induced mitophagy. In line with this, confocal microscopy demonstrated the co-localization between mitochondria and lysosomes labeled with the specific lysosomal fluorescent staining dye LysoTracker Green DND-26 (referred to as LysoTracker) in the live Nsp14-overexpressed cells (Manders’ overlap coefficient increased from 0.32 to 0.84, Fig. 3F). Moreover, the Nsp14-overexpressed cells were examined for mitophagy using transmission electron microscopy (TEM). As depicted in Fig. 3G, Nsp14 overexpression led to the disappearance of cristae structures in certain mitochondria, which were wrapped by autophagosomes (vesicular-like structures), showing characteristic features of mitophagy. These results provide evidence that PEDV Nsp14 induces mitophagy.

### PEDV Nsp14 interacts with NDP52 and TOM20

**Fig 4:**
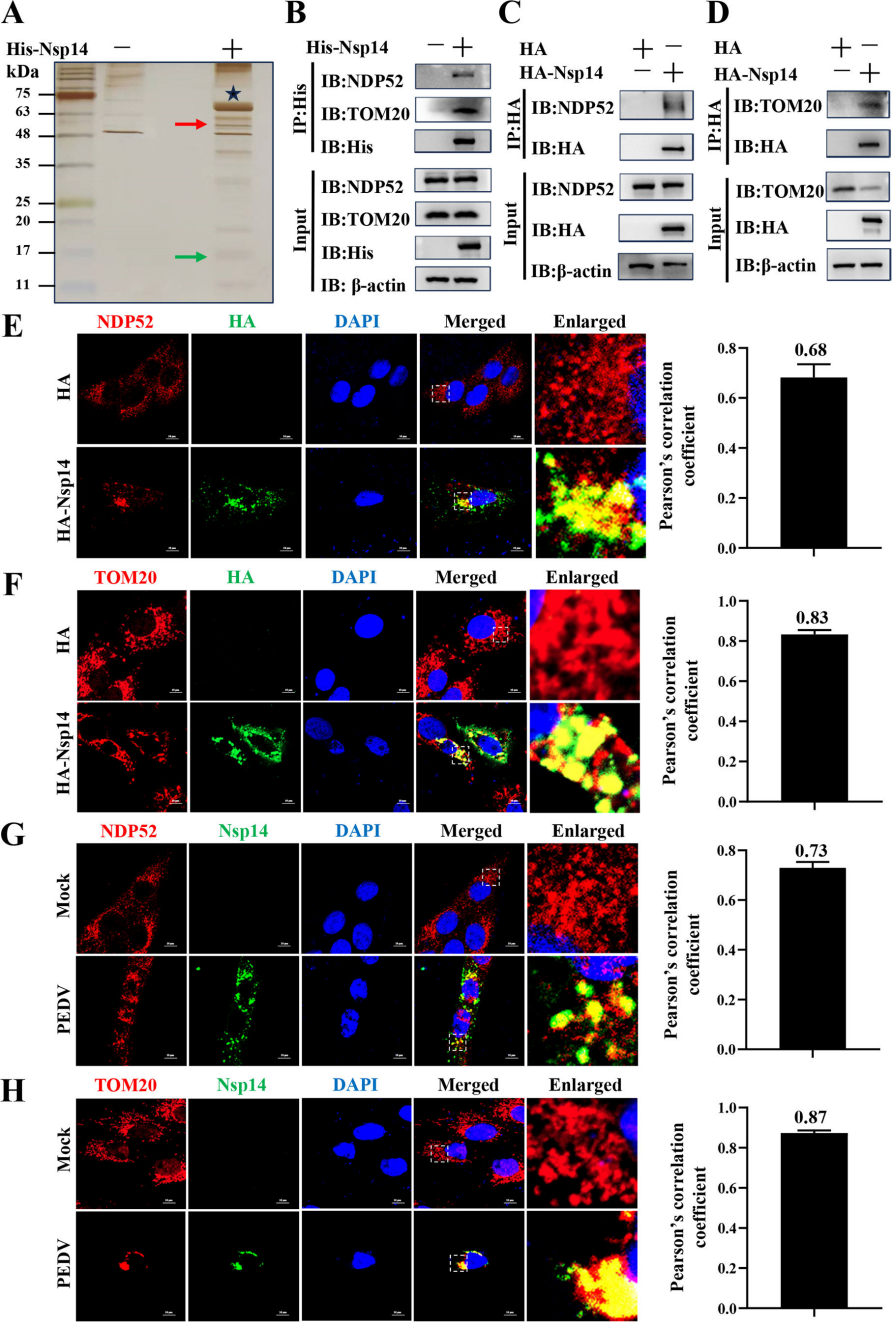
PEDV Nsp14 interacts with NDP52 and TOM20. (**A, B**) The Ni-NTA agaroses were bound by His-Nsp14 and incubated with IPEC-J2 WCLs at 4°C overnight. The resulting complexes were separated using SDS-PAGE and subjected to silver staining or WB analysis with the specific antibodies. The asterisk marked the bait protein His-Nsp14, the red arrow denoted NDP52, and the green arrow pointed to TOM20. (**C, D**) IPEC-J2 cells were transfected with the pCAGGS-HA-Nsp14 plasmid or HA-tagged empty vector for 36 h, with the HA-tagged empty vector serving as a negative control. IP was performed with anti-HA magnetic beads, and WB was conducted with the specific antibodies. (**E, F**) IPEC-J2 cells were transfected with the pCAGGS-HA-Nsp14 plasmid or HA-tagged empty vector, and fluorescence signals were visualized with the specific antibodies using confocal microscopy at 36 hpt. The assessment of interaction was conducted by calculating the Pearson’s correlation coefficient using the JaCoP plugin in the ImageJ software. Scale bars = 10 µm. (**G, H**) IPEC-J2 cells were infected with PEDV (0.2 MOI) for 36 h. Fluorescence signals were visualized with the specific antibodies using confocal microscopy. The assessment of interaction was conducted by calculating the Pearson’s correlation coefficient using the JaCoP plugin in the ImageJ software. Scale bars = 10 µm.

To explore the mechanisms involved in PEDV Nsp14-induced mitophagy, we conducted a pull-down assay coupled with liquid chromatography and tandem mass spectrometry (LC-MS/MS) using His-Nsp14 as bait. In brief, recombinant His-Nsp14 was expressed in *Escherichia coli* BL21 and then loaded on Ni-NTA agarose to capture endogenous host cell proteins. The captured proteins were subsequently separated by 12.5% sodium dodecyl sulfate-polyacrylamide gel electrophoresis (SDS-PAGE) and visualized using silver staining. The most prominent differential protein bands on SDS-PAGE were selected for further analysis by LC-MS/MS and identified as NDP52 marked by the red arrow and TOM20 marked by the green arrow (Fig. 4A). In parallel with these identifications, the capture of NDP52/TOM20 by His-Nsp14 was validated by WB (Fig. 4B). We further demonstrated that Nsp14 interacted with endogenous NDP52 or TOM20 in the Nsp14-overexpressed IPEC-J2 cells by immunoprecipitation (IP; Fig. 4C and D) and confocal microscopy (the Pearson’s correlation coefficients were 0.68 and 0.83, Fig. 4E and F), respectively. Moreover, we monitored the interaction between Nsp14 and NDP52 or TOM20 in the PEDV-infected cells (the Pearson’s correlation coefficients were 0.73 and 0.87, Fig. 4G and H). These findings collectively corroborate that PEDV Nsp14 interacts with both NDP52 and TOM20.

### PEDV Nsp14 recruits NDP52 to mitochondria to induce mitophagy

**Fig 5:**
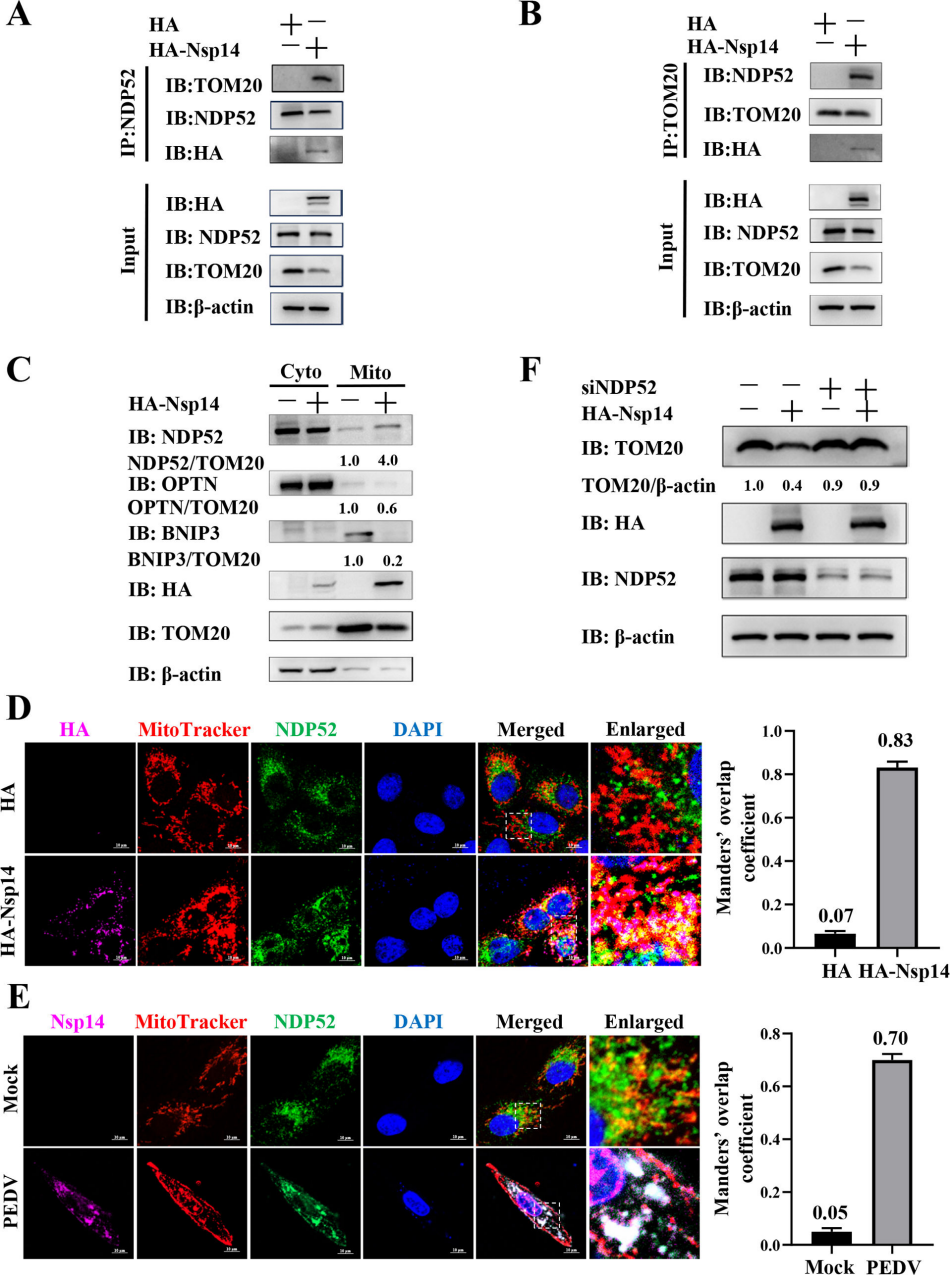
PEDV Nsp14 recruits NDP52 to mitochondria to induce mitophagy. (**A, B**) IPEC-J2 cells were transfected with the pCAGGS-HA-Nsp14 plasmid or HA-tagged empty vector for 36 h, followed by IP using protein G magnetic beads pre-incubated with anti-NDP52 or anti-TOM20 pAbs, and subsequent WB analysis. (**C**) IPEC-J2 cells were transfected with the pCAGGS-HA-Nsp14 plasmid or HA-tagged empty vector for 36 h. The cytoplasmic and mitochondrial fractions were isolated for WB analysis with the indicated antibodies. (**D**) IPEC-J2 cells were transfected with the pCAGGS-HA-Nsp14 plasmid or HA-tagged empty vector for 36 h. The cells were subjected to confocal microscopy using the specific antibodies and fluorescent reagents. The assessment of co-localization was conducted by calculating the Manders’ overlap coefficient using the JaCoP plugin in the ImageJ software. Scale bars = 10 µm. (**E**) IPEC-J2 cells were infected with or without PEDV for 36 h. The cells were subjected to confocal microscopy using the specific antibodies and fluorescent reagents. The assessment of co-localization was conducted by calculating the Manders’ overlap coefficient using the JaCoP plugin in the ImageJ software. Scale bars = 10 µm. (**F**) IPEC-J2 cells were transfected with siNC or siNDP52. At 24 hpt, the cells were transfected with the pCAGGS-HA-Nsp14 plasmid or HA-tagged empty vector, followed by WB.

NDP52 has been identified as a crucial mitophagy receptor, and its recruitment to mitochondria induces mitophagy (34, 36–38). As a result, we speculated that Nsp14 mediated the interaction between NDP52 and TOM20, which recruited NDP52 to mitochondria to induce mitophagy. To prove this speculation, we first conducted IP to test the interaction between NDP52 and TOM20 and found that NDP52 did not interact with TOM20 in the absence of Nsp14 (Fig. 5A and B). However, Nsp14 overexpression mediated their interaction (Fig. 5A and B). To further validate the recruitment of NDP52 on mitochondria by Nsp14, we isolated the mitochondria from the Nsp14-overexpressed cells and analyzed the protein levels of NDP52 by WB. The results showed that Nsp14 did not increase the protein levels of other mitophagy receptors, including OPTN and BNIP3, while specifically increasing that of NDP52 on mitochondria (Fig. 5C). Confocal microscopy also confirmed that both Nsp14 overexpression and PEDV infection recruited NDP52 on mitochondria (Fig. 5D and E). Moreover, we applied the small interfering RNA (siRNA) targeting NDP52 (siNDP52) to examine its effect on Nsp14-induced mitophagy. As shown in Fig. 5F, *NDP52* knockdown reversed the Nsp14-induced decrease in TOM20 abundance. These results reveal that PEDV Nsp14 recruits NDP52 to mitochondria by mediating its interaction with TOM20, which induces mitophagy.

### PEDV Nsp14 induces NDP52-mediated mitophagy to degrade MAVS for suppressing IFN-β production

**Fig 6:**
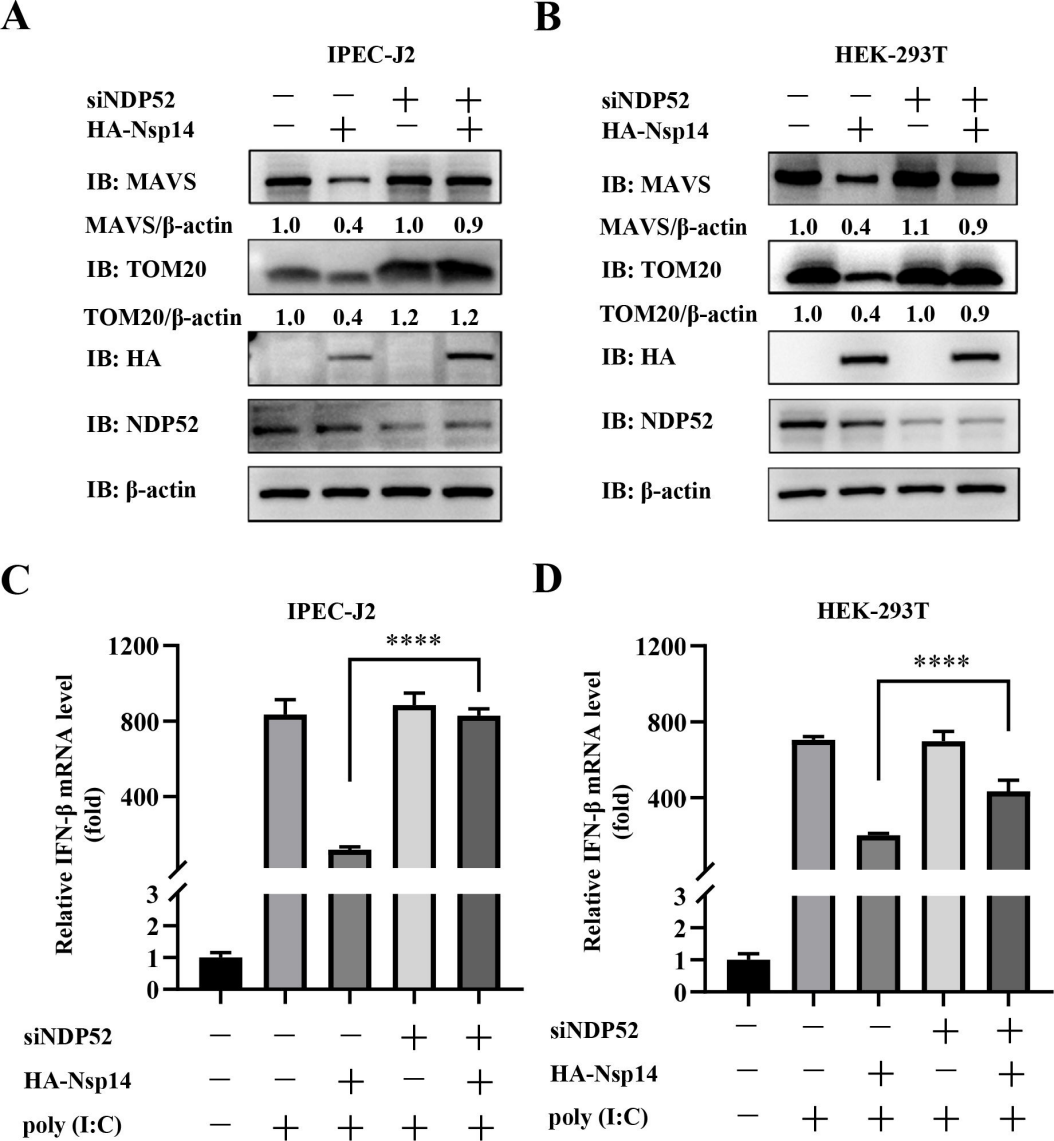
PEDV Nsp14 induces NDP52-mediated mitophagy to degrade MAVS for suppressing IFN-β production. (**A, B**) IPEC-J2 cells and HEK-293T cells with or without *NDP52* knockdown were transfected with either the pCAGGS-HA-Nsp14 plasmid or HA-tagged empty vector for 36 h. The WCLs were analyzed by WB. (**C, D**) IPEC-J2 cells and HEK-293T cells with or without *NDP52* knockdown were transfected with either the pCAGGS-HA-Nsp14 plasmid or HA-tagged empty vector, and then transfected by poly (I:C). RT-qPCR was applied to evaluate the mRNA levels of IFN-β. The cells transfected with siNC and without poly (I:C) served as a negative control. Data represent means ± SEM from three independent experiments. Statistical analysis was carried out using Student’s *t* test. ****P < 0.0001.

Based on the above results, we examined whether Nsp14 promoted the degradation of MAVS via NDP52-mediated mitophagy to inhibit IFN-β production in IPEC-J2 and human embryonic kidney 293T (HEK-293T) cells. As shown in Fig. 6A and B, Nsp14 overexpression led to degradation of both MAVS and TOM20, while *NDP52* knockdown inhibited their degradation, which indicated that Nsp14-induced mitophagy degraded MAVS. We further investigated the impact of Nsp14-induced mitophagy on IFN-I production. RT-qPCR analyses revealed that Nsp14 overexpression inhibited poly (I:C)-induced IFN-β production. However, this inhibitory effect was significantly attenuated in the *NDP52*-knockdown cells (Fig. 6C and D). These findings show that Nsp14 induces NDP52-mediated mitophagy to degrade MAVS and inhibit IFN-β production.

### PEDV Nsp14 induces mitophagy-mediated degradation of MAVS to inhibit IFN-β production and facilitate viral proliferation during infection

**Fig 7:**
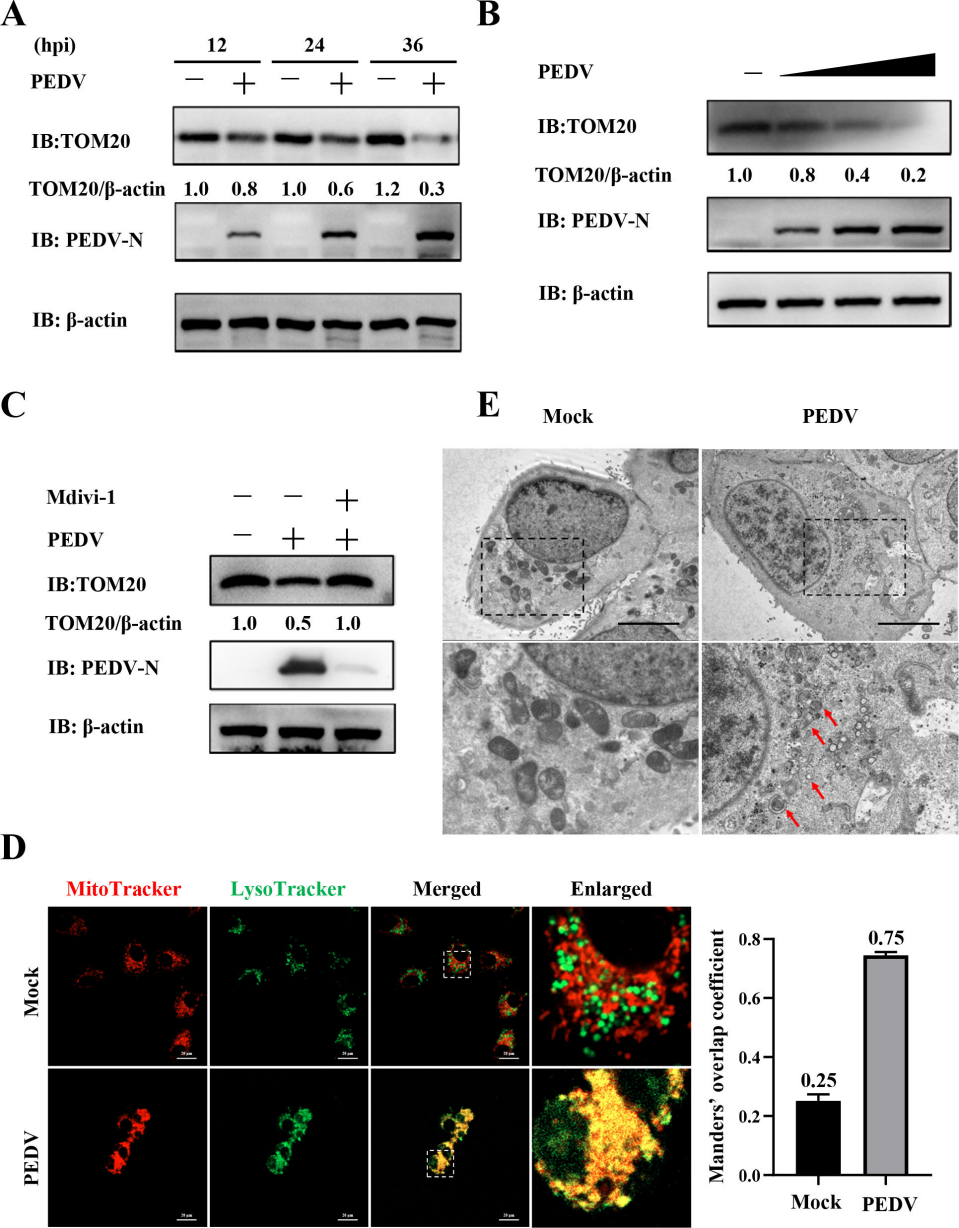
PEDV induces mitophagy in IPEC-J2 cells. (**A**) IPEC-J2 cells were infected with or without 0.2 MOI PEDV. WCLs were prepared at 12, 24, and 36 hpi for WB analysis. (**B**) IPEC-J2 cells were infected with PEDV at different MOIs (0.1, 0.2, and 0.4 MOI) or mock-infected for 36 h. WCLs were analyzed by WB. (**C**) IPEC-J2 cells were infected with 0.2 MOI PEDV for 6 h, and then treated with 5 µM Mdivi-1 for 30 h. WCLs were analyzed using WB. (**D**) IPEC-J2 cells were infected with or without 0.2 MOI PEDV and stained using MitoTracker (red) and LysoTracker (green). The fluorescent signals in live cells were visualized using confocal microscopy. The assessment of co-localization was conducted by calculating the Manders’ overlap coefficient using the JaCoP plugin in the ImageJ software. Scale bars = 20 µm. (**E**) IPEC-J2 cells were infected with or without PEDV (0.2 MOI) for 36 h and fixed for TEM (scale bars = 5 µm). Red arrows indicate the mitochondria engulfed by autophagosomes.

**Fig 8:**
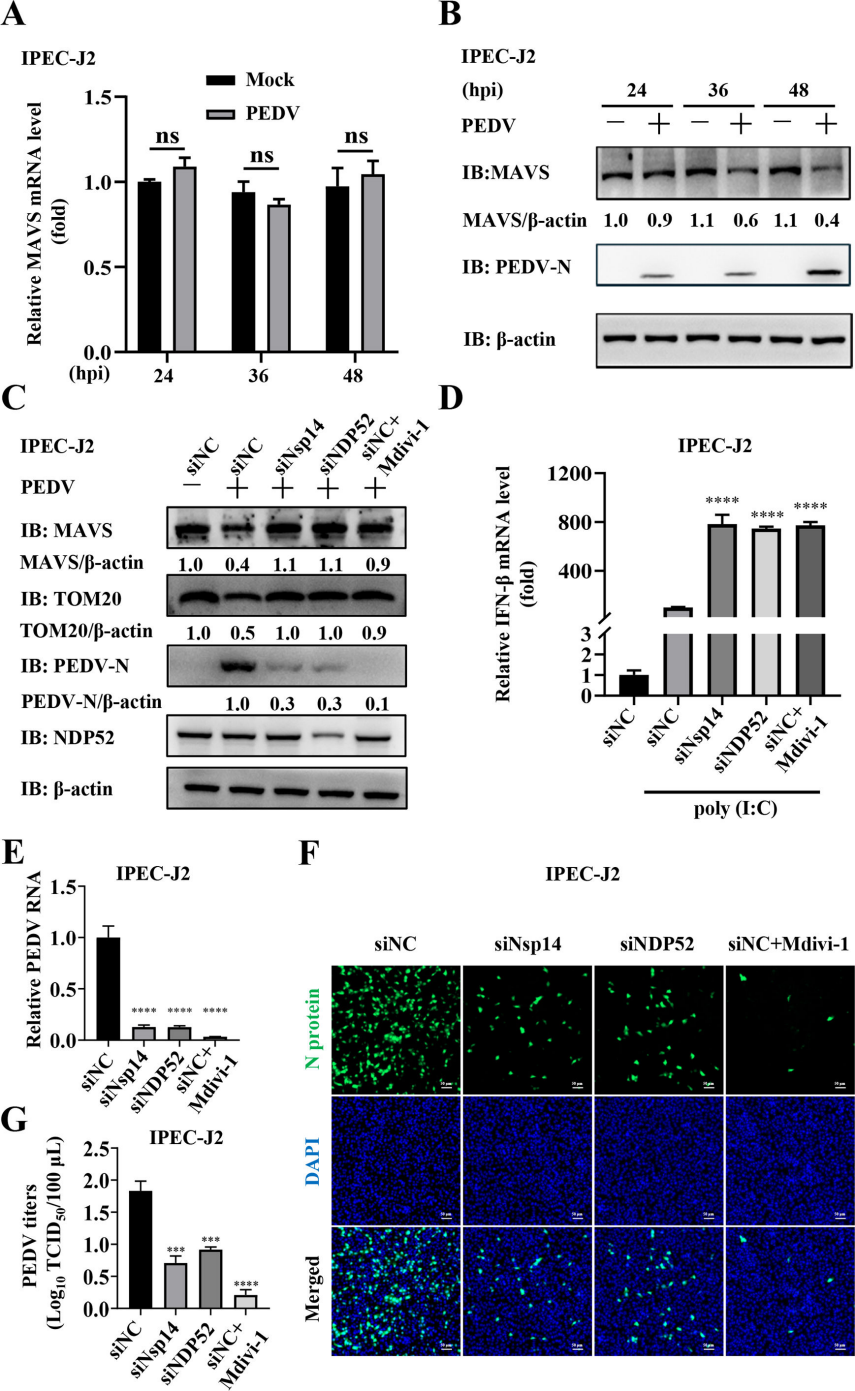
PEDV Nsp14 induces mitophagy-mediated degradation of MAVS to inhibit IFN-β production and facilitate viral proliferation during infection in IPEC-J2 cells. (**A, B**) IPEC-J2 cells were infected with or without 0.2 MOI PEDV and collected at the indicated time points (24, 36, and 48 hpi). The MAVS mRNA and protein levels were detected by RT-qPCR and WB, respectively. (**C through G**) IPEC-J2 cells were transfected with siNC, siNsp14, or siNDP52. At 24 hpt, the cells were infected with 0.2 MOI PEDV. In parallel, Mdivi-1 was applied as a control. The MAVS, TOM20, and PEDV N protein abundance were analyzed by WB (**C**). After poly (I:C) transfection, the mRNA levels of IFN-β were detected by RT-qPCR. The cells transfected with siNC alone served as control (**D**). RT-qPCR was used to evaluate the PEDV mRNA levels (**E**). PEDV infectivity was detected using IFA with the mouse anti-PEDV N protein mAb. The cells were stained with DAPI for nuclear staining. Scale bars = 50 µm (**F**). PEDV titers were measured by assessing TCID_50_ (**G**). Data represent means ± SEM from three independent experiments. Statistical analysis was carried out using one-way ANOVA or Student’s *t* test. ns *P >* 0.05, ***P < 0.001 and ****P < 0.0001.

Next, we detected whether Nsp14 induced mitophagy to degrade MAVS and inhibit IFN-β production during PEDV infection. We first validated that PEDV infection triggered mitophagy in IPEC-J2 cells. As shown in Fig. 7A and B, a significant reduction in TOM20 abundance was observed in the PEDV-infected cells, whereas the PEDV-induced decrease in TOM20 abundance was effectively inhibited by Mdivi-1 (Fig. 7C). Furthermore, live-cell confocal microscopy revealed pronounced co-localization between mitochondria and lysosomes upon PEDV infection (Manders’ overlap coefficient increased from 0.25 to 0.75, Fig. 7D), and TEM showed that numerous mitochondria were enclosed within autophagosomes in the PEDV-infected cells (Fig. 7E). As expected, both the siRNA targeting PEDV Nsp14 (siNsp14) and siNDP52 suppressed PEDV-induced mitophagy as indicated by attenuated TOM20 degradation, similar to Mdivi-1 treatment (Fig. 8C). Thereafter, we analyzed the MAVS mRNA and protein levels during PEDV infection by RT-qPCR and WB, respectively. The results indicated that PEDV infection did not affect the MAVS mRNA abundance (Fig. 8A), while decreasing its protein levels in a time-dependent manner (Fig. 8B). By contrast, siNsp14, siNDP52, and Mdivi-1 all inhibited PEDV-induced MAVS degradation (Fig. 8C) and enhanced IFN-β production (Fig. 8D). We subsequently investigated the impact of Nsp14-mediated mitophagy on PEDV proliferation. SiNsp14, siNDP52, and Mdivi-1 all decreased PEDV RNA levels as detected by RT-qPCR (Fig. 8E) and N protein expression as detected by WB (Fig. 7C and 8C), respectively. Indirect immunofluorescence assay (IFA) also exhibited that siNsp14, siNDP52, and Mdivi-1 exerted significant inhibitory effects on PEDV infectivity (Fig. 8F). More importantly, assessment of 50% tissue culture infected dose (TCID_50_) showed that siNsp14, siNDP52, and Mdivi-1 lowered PEDV titers by at least 10-fold (> 1 log_10_ TCID_50_/100 µL, Fig. 8G).

**Fig 9:**
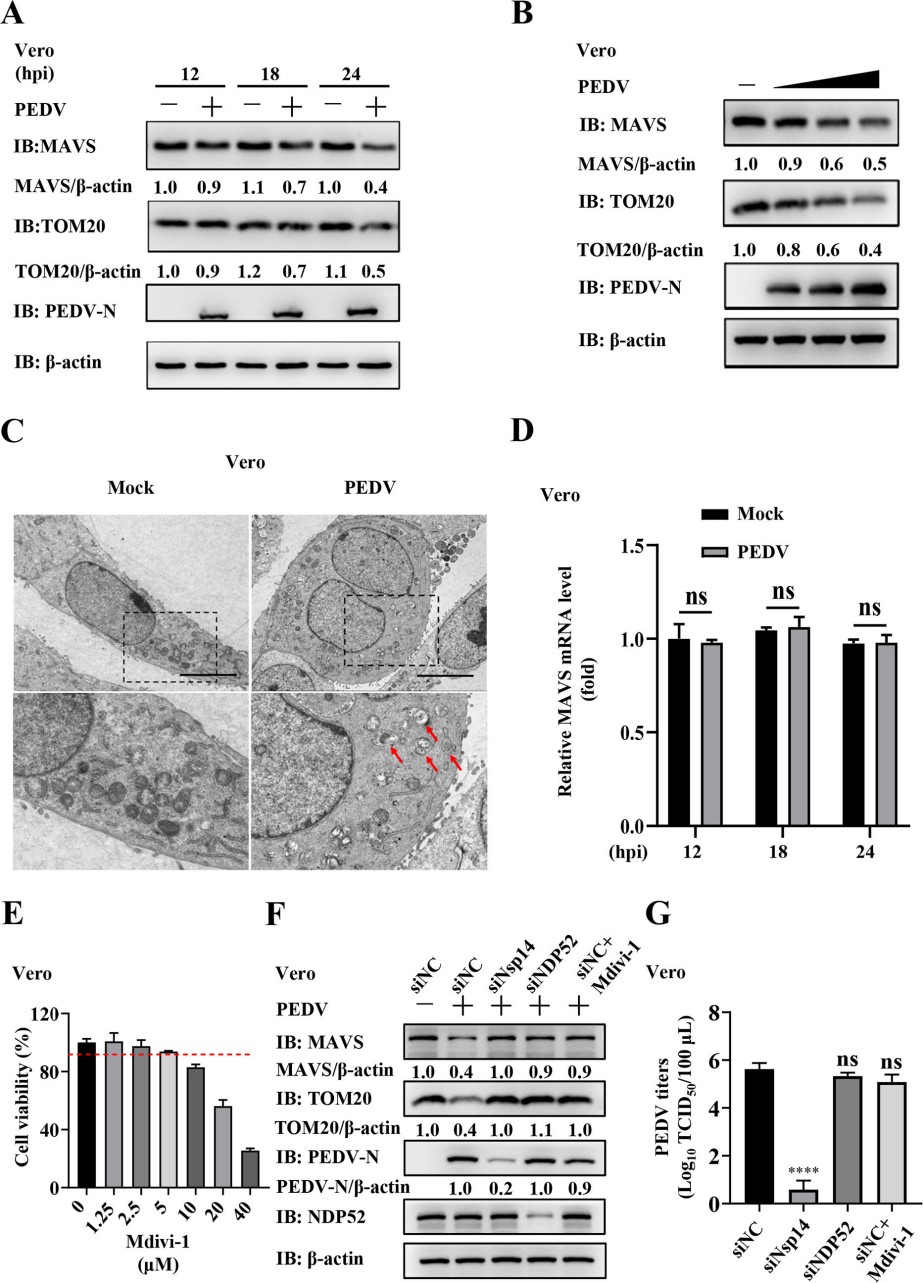
PEDV-induced mitophagy and MAVS degradation had no effect on viral proliferation in Vero cells. (**A**) Vero cells were infected with or without 0.2 MOI PEDV and collected at the indicated time points (12, 18, and 24 hpi). The MAVS and TOM20 protein levels were analyzed by WB. (**B**) Vero cells were infected with PEDV at different MOIs (0.1, 0.2, and 0.4 MOI) or mock-infected for 24 h. WCLs were analyzed by WB. (**C**) Vero cells were infected with or without PEDV (0.2 MOI) for 18 h and fixed for TEM (scale bars = 5 µm). (**D**) Vero cells were infected with or without 0.2 MOI PEDV and collected at the indicated time points (12, 18, and 24 hpi). The MAVS mRNA levels were detected by RT-qPCR. (**E**) The cytotoxicity of Mdivi-1 on Vero cells was detected using the cell viability assay. (**F, G**) Vero cells were transfected with siNC, siNsp14, or siNDP52. At 24 hpt, the cells were infected with 0.2 MOI PEDV. In parallel, Mdivi-1 was applied as a control. The MAVS, TOM20, and PEDV N protein abundance were analyzed by WB (**F**). PEDV titers were measured by assessing TCID_50_ (**G**). The cells transfected with siNC alone served as a control. Data represent means ± SEM from three independent experiments. Statistical analysis was carried out using one-way ANOVA or Student’s *t* test. ns *P >* 0.05 and ****P < 0.0001.

To further verify the role of PEDV Nsp14-induced mitophagy during viral infection, we attempted to construct an Nsp14-deficient PEDV, but failed to rescue it (data not shown). Therefore, we performed the assays in Vero cells, which are defective in IFN production (39, 40). As shown in Fig. 9A through F, PEDV infection also induced mitophagy and MAVS degradation in Vero cells. Similarly, inhibition of PEDV-induced mitophagy by siRNAs and Mdivi-1 restored MAVS protein levels (Fig. 9F). However, inhibition of mitophagy and restoration of MAVS had no effect on PEDV proliferation (Fig. 9F and G).

These data demonstrate that PEDV Nsp14 induces mitophagy-mediated degradation of MAVS and subsequent inhibition of IFN-I production to facilitate viral proliferation.

## DISCUSSION

Mitochondria play pivotal roles in various cellular processes, such as energy metabolism (18), cell apoptosis (41, 42), intracellular Ca^2+^ homeostasis (43), and innate immunity (44). Mitochondria face various types of stresses, such as oxidative stress (45), cellular protein aggregation (46), and mitochondrial DNA damage (47), which cause mitochondrial damage and dysfunction. Damaged and dysfunctional mitochondria are eliminated by a selective autophagy called mitophagy (48). An increasing amount of evidence shows that certain viruses suppress host innate immunity via mitophagy to promote their proliferation (49). In this study, we have unraveled that PEDV suppresses host innate immune responses via mitophagy to facilitate self-proliferation.

Here, we confirmed that PEDV inhibited IFN-β production and identified PEDV Nsp14 as a potent inhibitor (Fig. 1). Previous studies have reported that PEDV Nsp14 suppresses IFN-β production (13, 28, 29). A recent study shows that PEDV Nsp14 inhibits IFN-β production by induction of RIG-I degradation via caspase pathways (50). However, the mechanisms involved in its immunosuppression remain to be comprehensively elaborated. In this study, we determined that MAVS was a target for Nsp14-mediated inhibition of IFN-β production and was degraded by Nsp14 via the autolysosomal pathway (Fig. 2). MAVS is located on the mitochondrial outer membrane and is a crucial adapter protein in transducing antiviral innate immune signals (51, 52). There is substantial evidence indicating that viruses inhibit host innate immune responses by targeting MAVS via modification or degradation (53). For example, RNA virus infections utilize the OTUD1-Smurf1 axis to promote MAVS-K48 ubiquitination-mediated degradation and inhibit the RLRs pathway (54).

Furthermore, we found that PEDV Nsp14 induced mitophagy to degrade MAVS (Fig. 3 and 6). In detail, we revealed that Nsp14 interacted with NDP52/TOM20 through multiple methods (Fig. 4). NDP52, also known as calcium-binding and coiled-coil domain 2, belongs to the nuclear dot protein family (55). NDP52 is often classified as a ubiquitin-dependent selective autophagy receptor along with P62, NBR1, OPTN, and TAX1BP1 (56) and is also a member of mitophagy receptors (57, 58). NDP52 has been reported to be exploited by viruses to enhance their replication. For instance, coxsackievirus B3 and classical swine fever virus induce mitophagy via the NDP52-mediated ubiquitin-dependent pathway to inhibit innate immunity and promote viral proliferation (59, 60). NDP52 is also demonstrated to be recruited to mitochondria and thereby triggers mitophagy under non-ubiquitin binding conditions (36, 38). In the current study, we detected that PEDV Nsp14 mediated the interaction between NDP52 and TOM20, which recruited NDP52 to mitochondria to induce mitophagy (Fig. 5).

Next, we determined that Nsp14 induced mitophagy-mediated degradation of MAVS to impair IFN-β production (Fig. 6). More importantly, we corroborated that Nsp14 induced mitophagy during PEDV infection (Fig. 7 and 8). We further confirmed that Nsp14 induced mitophagy-mediated degradation of MAVS to antagonize IFN-β production and facilitate viral proliferation in IPEC-J2 cells (Fig. 8). Moreover, we found that PEDV Nsp14-induced mitophagy and degradation of MAVS had no effect on viral proliferation in IFN production-deficient Vero cells (Fig. 9). All these data show that Nsp14-induced mitophagy is important for regulating IFN-β production and viral propagation during PEDV infection. We actually made efforts to construct an Nsp14-deficient PEDV, but found that the deletion was lethal for the virus, indicating that Nsp14 is critical for PEDV viability. Previous studies have also shown that Nsp14 is essential for coronaviruses (61, 62). We will identify the specific residues responsible for Nsp14-induced mitophagy and generate site-mutated PEDV to verify their impacts in the future. In addition, we plan to validate these *in vitro* results *in vivo* using the wild-type and mutant PEDV.

In fact, mitophagy is currently the focus of research as it is hijacked by viruses for their own benefit (63). For instance, the matrix protein of human parainfluenza virus type 3 and the PB1-F2 protein of influenza A virus both interact with Tu translation elongation factor to trigger mitophagy and degrade MAVS, thus inhibiting innate immune responses (24, 25). In addition, the ORF10 protein of severe acute respiratory syndrome coronavirus 2 interacts with NIX (BCL2 interacting protein 3-like) to induce mitophagy and degrade MAVS, leading to inhibition of IFN-I production (26). Therefore, mitophagy is a promising target for developing broad-spectrum antiviral agents.

**Fig 10:**
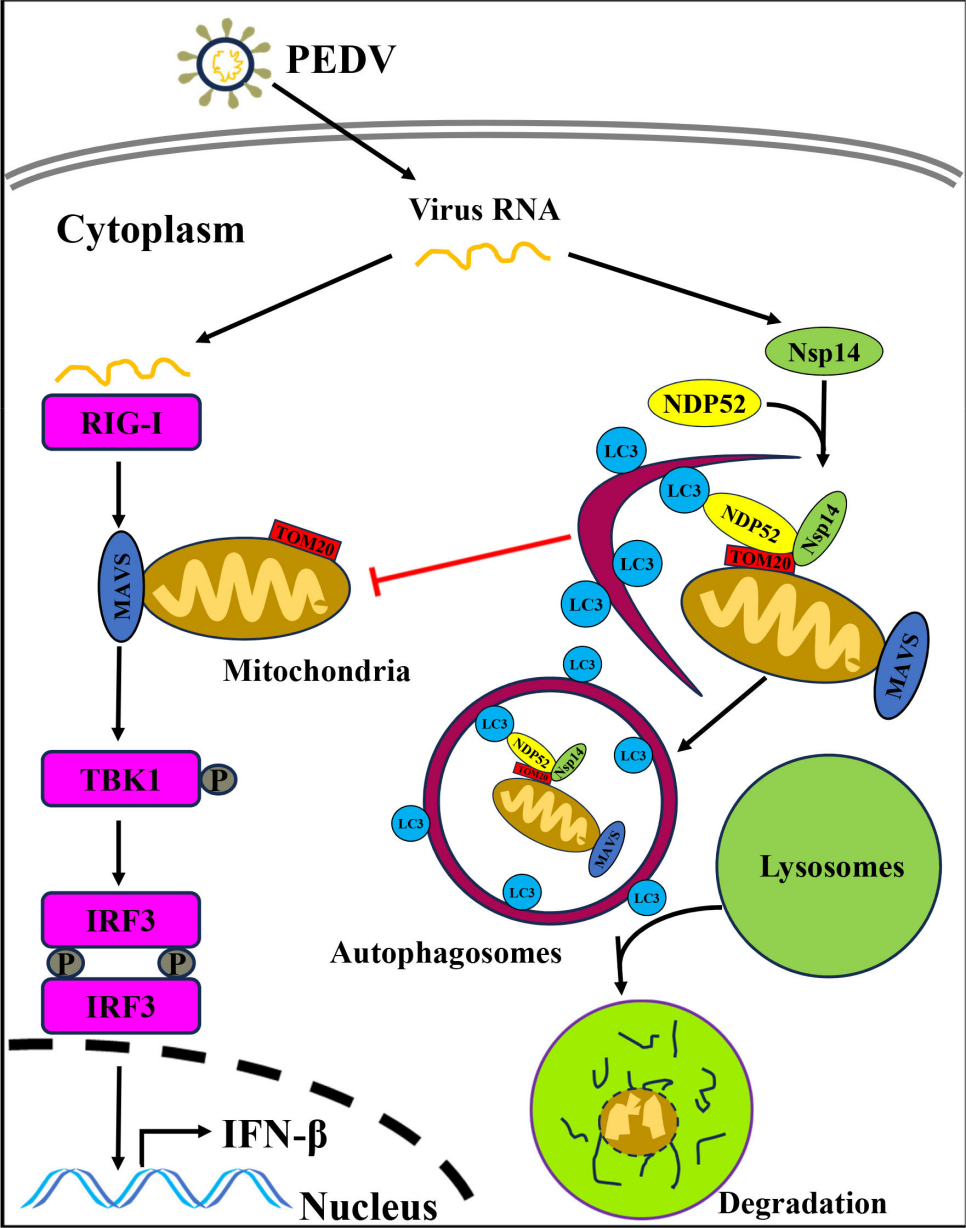
A proposed model depicts that PEDV Nsp14 induces mitophagy to degrade MAVS for suppressing IFN-β production and facilitating viral proliferation. Mechanistically, PEDV Nsp14 mediates the interaction between NDP52 and TOM20, which leads to the recruitment of NDP52 to mitochondria. This process induces mitophagy, which degrades MAVS and inhibits the IFN-β production, ultimately promoting virus proliferation.

Based on our results, we propose a model to elucidate the underlying mechanism by which PEDV Nsp14 induces mitophagy to inhibit IFN-β production and promote viral proliferation (Fig. 10). During PEDV infection, Nsp14 mediates the interaction between NDP52 and TOM20 and results in the recruitment of NDP52 to mitochondria. This process induces mitophagy to degrade MAVS and thereby inhibits IFN-β production, which promotes viral proliferation.

In conclusion, our study demonstrates that PEDV Nsp14 triggers mitophagy to degrade MAVS and facilitate viral proliferation. These results deepen our understanding of PEDV pathogenesis. More importantly, considering its significance in various viral infections, mitophagy can be targeted for broad-spectrum anti-viral therapies.

## MATERIALS AND METHODS

### Cell culture and virus propagation

IPEC-J2, Vero, and HEK-293T cells were maintained in our laboratory (64). These cells were cultured in Dulbecco’s modified Eagle’s medium (DMEM, 12100; Solarbio, Beijing, China) supplemented with 10% heat-inactivated fetal bovine serum (FBS, 10270-106; Gibco, Waltham, USA) and antibiotics (penicillin 100 U/mL and streptomycin 100 mg/mL; P1400, Solarbio, Beijing, China) under humidified conditions at 37°C with 5% CO_2_.

The PEDV CH/hubei/2016 strain (GenBank: KY928065.1) was kept in our laboratory (65). To propagate the virus, PEDV was inoculated into DMEM supplemented with 6 µg/mL trypsin (T4799; Sigma-Aldrich, St. Louis, USA) to facilitate infection of Vero cells.

### Antibodies

Rabbit anti-TOM20 polyclonal antibodies (pAbs, 11802-1-AP), horseradish peroxidase (HRP)-labeled mouse anti-β-actin monoclonal antibody (mAb, HRP-66009), rabbit anti-NDP52 pAbs (12229-1-AP), and rabbit anti-OPTN pAbs (10837-1-AP) were purchased from Proteintech (Wuhan, China). Mouse anti-PEDV N protein mAb (PEDV 3F12, 9191) was purchased from MEDIAN Diagnostics (Chuncheon, Korea). Rabbit anti-BNIP3 mAb (CY6771) was purchased from Abways (Shanghai, China). Rabbit anti-HA mAb (3724T), rabbit anti-MAVS mAb (24930S), and rabbit isotype IgG (2729) were purchased from Cell Signaling Technology (Danvers, USA). Alexa Fluor 488 goat anti-mouse IgG H&L preadsorbed pAbs (ab150117) and Alexa Fluor 647 donkey anti-rabbit IgG H&L preadsorbed pAbs (ab150075) were purchased from Abcam (Cambridge, United Kingdom). HRP-labeled goat anti-mouse IgG pAbs (A21010), HRP-labeled goat anti-rabbit IgG pAbs (A21020), and HRP-labeled mouse anti-rabbit IgG light chain pAbs (A25022) were purchased from Abbkine (Wuhan, China).

### Reagents

RNAiso plus (9109) and PrimeScript RT master mix kit (RR036A) were purchased from Takara Bio (Dalian, China). 4′,6′-diamidino-2-phenylindole (DAPI, C0065), dimethyl sulfoxide (DMSO, D8371), and phosphate-buffered solution (PBS, P1010) were purchased from Solarbio. ChamQ universal SYBR qPCR master mix was purchased from Vazyme Biotech (Nanjing, China). Enhanced chemiluminescence reagent (ECL, P0013B) was purchased from NCM Biotech (Suzhou, China). The plasmids pCMV-MDA5-3×Flag-Neo (P37736), pCMV-RIG-I-3×Flag-Neo (P37735), pCMV-MAVS-3×Flag-Neo (P37501), pCMV-TBK1-3×Flag-Neo (P37737), pCMV-IRF3-3×Flag-Neo (P37466), and pCMV-3×Flag- Neo (P8196) were purchased from MiaoLing Biology (Wuhan, China). Protein G magnetic beads (HY-K0204), anti-HA magnetic beads (HY-K0201), CQ (HY-17589A), BafA1 (HY-100558), Mdivi-1 (HY-15886), MG132 (HY-13259), polyfast transfection reagent (HY-K1014), and poly (I:C) (HY-107202) were purchased from MedChemExpress (South Brunswick, USA). Cell counting kit-8 (CCK-8, GK10001) was purchased from GLPBIO Technology (Montclair, USA). 4% paraformaldehyde (PFA, P0099), WB/IP lysis buffer (P0013), phenylmethanesulfonyl fluoride (PMSF, ST506-2), enhanced BCA protein assay kit (P0010), and Triton X-100 (P0096) were purchased from Beyotime Biotechnology (Shanghai, China). Lipofectamine RNAiMAX transfection reagent (13778150), MitoTracker (m7512), and LysoTracker (L7526) were purchased from Invitrogen (Carlsbad, USA). TransIntro PL transfection reagent (FT301-01) was purchased from TransGen Biotech (Beijing, China). Ni-NTA agaroses (70666-4) and 0.22 µm polyvinylidene fluoride membranes (PVDF, ISEQ00010) were purchased from Merck Millipore (Billerica, USA). 3-MA (M9281), complete Freund’s adjuvant (F5881), and incomplete Freund’s adjuvant (F5506) were purchased from Sigma-Aldrich. Protease inhibitor cocktail (04693116001) was purchased from Roche (Basel, Switzerland). 2.5% glutaraldehyde fixative (G1102) was purchased from Servicebio (Basel, Switzerland). LR white resin (G1102) was purchased from HaideBio (Beijing, China). The minute mitochondria isolation kit (MP007) was purchased from Invent Biotechnologies (Eden Prairie, USA). The 12.5% SDS-PAGE kit (PG113) was purchased from Epizyme (Shanghai, China).

### Expression vector construction and transfection

The genes encoding Nsp1-Nsp16 (excluding Nsp11), S, E, M, and N proteins were synthesized by Genewiz Company (Suzhou, China) and incorporated into the pCAGGS vector with HA tags at the N-termini. The expression vectors were transfected into cells using TransIntro PL transfection reagent according to the manufacturer’s instructions. Otherwise specified, 24-well cell culture plates were transfected with 0.5 µg of each plasmid per well, and six-well cell culture plates received a transfection of 2 µg of each plasmid per well.

### RT-qPCR

Total RNA was extracted using the RNAiso Plus reagent and reverse transcribed into cDNA using the PrimeScript RT master mix kit. The cDNA was amplified by PCR using the ChamQ universal SYBR qPCR master mix on an RT-qPCR system (7500 Fast, Applied Biosystems; Foster City, USA). β-actin served as the internal reference for normalizing mRNA abundance, and the 2^−ΔΔCT^ method was applied to evaluate the mRNA levels of various target proteins (66). The primers used for RT-qPCR are listed in Table 1.

**TABLE 1:** Primers for RT-qPCR used in this study

Name	5′−3′ (sense)	5′−3′ (antisense)
MAVS (pig)	GGACCTCTTCGACAGCCTTC	GCTGTTTGAATTCCGCAGCA
IFN-β (pig)	GCAGTATTGATTATCCACGAGA	TCTGCCCATCAAGTTCCAC
β-actin (pig)	GAATCCTGCGGCATCCACGA	CTCGTCGTACTCCTGCTTGCT
IFN-β (human)	GGACAGGATGAACTTTGACA	AGACATTAGCCAGGAGGTT
β-actin (human)	GGAAATCGTGCGTGACAT	AAGGAAGGCTGGAAGAGT
MAVS (monkey)	AGTGCCTACCACCTTGATGC	GGATGGTGCTGGATTGGTGA
β-actin (monkey)	CAACCTTCCTTCCTGGGCAT	CTGTGTTGGCGTACAGGTCT

### Western blot

The cells were washed three times with PBS and subsequently lysed on ice for 30 min using WB/IP lysis buffer supplemented with the protease inhibitor PMSF. After centrifugation at 12,000 × *g* at 4°C for 10 min, the resulting supernatant was collected as whole cell lysate (WCL). The protein concentration of WCL was determined using an enhanced BCA protein analysis kit. Subsequently, the samples were separated by 12.5% SDS-PAGE and transferred onto PVDF membranes. The PVDF membranes were immersed in 5% skim milk at room temperature (RT) for 2 h, followed by incubation with the specific primary antibodies at 4°C overnight, and then incubated with the specific secondary antibodies at RT for 1 h. After washing, WB results were visualized with ECL reagent and imaged on a chemiluminescence imaging system (Vilber Fusion FX7, VILBER; Paris, France).

### Immunoprecipitation

The cells were transfected with the pCAGGS-HA-Nsp14 plasmid for 36 h. After cell lysis in WB/IP lysis buffer containing the protease inhibitor cocktail, the supernatant was obtained through centrifugation at 12,000 × *g* for 10 min. Anti-HA magnetic beads (30 µL) were subsequently incubated with the WCLs at RT for 2 h according to the instructions. In addition, after protein G magnetic beads were incubated with anti-NDP52 pAbs, anti-TOM20 pAbs, or isotype IgG at RT for 1 h, the beads were added to the WCLs and incubated at 4°C overnight. The precipitated immunocomplexes were collected using a magnetic holder and washed with PBST (PBS with Tween-20) more than six times. The associated proteins were tested by WB using the indicated antibodies.

### Cell viability assay and inhibitor treatments

The cytotoxicity of 3-MA, CQ, BafA1, MG132, and Mdivi-1 was evaluated using the CCK-8 kit according to the manufacturer’s protocol. Cells were treated with each inhibitor for 36 h, followed by CCK-8 solution incubation at 37°C for 2 h in 5% CO_2_. The optical density values were measured at 450 nm using a microplate reader (415-1577, Ortenberg, Germany) (67).

### Confocal microscopy and IFA

The cells were washed three times with pre-chilled PBS and subsequently fixed with 4% PFA at RT for 15 min. Following the removal of 4% PFA using PBS, permeabilization was performed using 0.1% Triton X-100 at RT for 10 min. Subsequently, the cells were blocked with PBS containing 5% bovine serum albumin at RT for 2 h, followed by incubation with the specific primary antibodies at 4°C overnight. After washing with PBS, appropriate fluorescent secondary antibodies were applied and incubated for 1.5 h. The cells were stained with DAPI for nuclear staining for 10 min. Finally, fluorescence images were acquired utilizing a confocal laser scanning microscope (LSM800, Carl Zeiss AG; Oberkochen, Germany) for confocal microscopy or IFA. The co-localization analyses were conducted utilizing the JaCoP plugin in the ImageJ software according to the established research protocols (68–70). Manders’ overlap coefficient (>0.6) suggests an actual overlap between the signals and is commonly used to represent the true degree of co-localization. Pearson’s correlation coefficient (>0.5) describes the correlation of the intensity distribution between channels (71, 72).

### TEM

The cells were collected and fixed using 2.5% glutaraldehyde fixative at RT for 30 min in the absence of light. Subsequently, the fixed cells underwent dehydration with a graded series of ethanol and were finally embedded in pure LR white resin at 4°C using BEEM capsules (69920-00, Electron Microscopy Sciences; Hatfield, USA). After polymerization with a low-temperature UV polymerizer (UVCC2515, Electron Microscopy; Beijing, China), the resin blocks were sliced into thin sections between 70 and 80 nm on an ultramicrotome (UC7, Leica; Wetzlar, Germany). These ultrathin sections were then transferred onto nickel grids with a 150-mesh Formvar film (FCF200-Cu-50, Electron Microscopy Sciences). Finally, the samples were observed under TEM (Ht7800/Ht7700, Hitachi; Tokyo, Japan) at an acceleration voltage of 80 kV.

### Pull-down assay and LC-MS/MS analysis

The cells were cultured for 24 h and subsequently lysed on ice for 30 min using WB/IP lysis buffer containing the protease inhibitor cocktail. After centrifugation at 4°C, 12,000 × *g* for 10 min, the supernatant was collected and reserved. The gene encoding PEDV Nsp14 was cloned into pET-32a (+) by Gentlegen (Suzhou, China), and the recombinant protein was expressed in *Escherichia coli* BL21-competent cells (CD601-02, TransGen; Beijing, China). The recombinant His-Nsp14 was purified by Ni-NTA agarose. Subsequently, the fresh Ni-NTA agaroses were incubated with the purified protein at RT for 2 h and washed six times with tris-buffered saline with Tween-20 (TBST). The Ni-NTA agaroses were further incubated with the supernatant at 4°C overnight, and then underwent six washes with TBST. The associated proteins were separated by 12.5% SDS-PAGE and then subjected to silver staining, followed by LC-MS/MS by Lumingbio (Shanghai, China).

### Preparation of pAbs against PEDV Nsp14

To generate mouse pAbs targeting Nsp14, 20 µg purified His-Nsp14 was mixed with either complete Freund’s adjuvant or incomplete Freund’s adjuvant and administered via intramuscular injection into female BALB/c mice aged 4-6 weeks every 2 weeks for three times. After three immunizations, blood was collected from the eye and prepared into serum for confocal microscopy. The experimental procedure utilized to generate pAbs was approved and supervised by the Ethical and Animal Welfare Committee of the Key Laboratory of Animal Immunology of the Ministry of Agriculture of China (Approval No. LLSC410230069).

### Isolation of mitochondria

Minute mitochondrial isolation kit was applied to isolate mitochondria according to the guidelines provided by the manufacturer. The mitochondria and cytoplasm were separated by differential centrifugation and used for subsequent WB analysis.

### RNA interference

SiRNA negative control (siNC), siNDP52, and siNsp14 were custom-designed and synthesized by GenePharma (Shanghai, China). The indicated siRNAs were transfected using Lipofectamine RNAiMAX according to the manufacturer’s instructions. The knockdown efficiency after transfection was validated. The specific siRNAs used are listed in Table 2.

**TABLE 2:** siRNAs used in this study

Gene	5′−3′ (sense)	5′−3′ (antisense)
siNsp14	GGUCAGUUCUUAAGUUAUATT	GGUCAGUUCUUAAGUUAUATT
siNDP52 (pig)	GGACGUCACAUGUCAUUAUTT	AUAAUGACAUGUGACGUCCTT
siNDP52 (human)	GCCCAAGGAUGAUGAGUAUTT	AUACUCAUCAUCCUUGGGCTT
siNC	UUCUCCGAACGUGUCACGUTT	ACGUGACACGUUCGGAGAATT

### PEDV titration assay

The infected cells underwent three freeze-thaw cycles and were subsequently centrifuged at 8,000 × *g* for 10 min to collect the supernatant. The supernatant was then serially diluted (10^−1^–10^−9^) in DMEM containing 6 µg/mL trypsin. The diluted virus supernatant was added to a 96-well plate seeded with Vero cells, which were washed three times with PBS. The plate was incubated at 37°C with 5% CO_2_ for 1 h following viral infection. Afterward, the cells were washed three times with PBS and added with fresh DMEM containing trypsin for further cultivation lasting 3–5 d. Cell cytopathic effects were quantified, and TCID_50_ values were calculated using the Reed-Muench method (73).

### Statistical analysis

Each experiment was conducted in triplicate and repeated independently at least three times. The data represent means of the three independent experiments ± the standard error of the mean (SEM). All statistical analyses and calculations were performed with the unpaired two-tailed Student’s *t* test or one-way analysis of variance (ANOVA) using GraphPad Prism 8.0 software (San Diego, USA). Statistical significance in the figures was indicated by asterisks (ns [not significant], *P < 0.05, **P < 0.01, ***P < 0.001, ****P < 0.0001).

## Data Availability

The data underlying this article will be shared on reasonable request to the corresponding author.
